# Remote Sensing for Crop Water Management: From ET Modelling to Services for the End Users

**DOI:** 10.3390/s17051104

**Published:** 2017-05-11

**Authors:** Alfonso Calera, Isidro Campos, Anna Osann, Guido D’Urso, Massimo Menenti

**Affiliations:** 1 GIS and Remote Sensing Group, Institute for Regional Development, University of Castilla-La Mancha, Campus Universitario SN, 02071 Albacete, Spain; isidro.campos@uclm.es (I.C.); anna.osann@gmail.com (A.O.); 2 Department of Agricultural Sciences, University of Naples Federico II, Via Università 100, 80055 Portici (NA), Italy; durso@unina.it; 3 Department of Geoscience and Remote Sensing, Delft University of Technology, Stevinweg 1, 2628 CN Delft, The Netherlands; M.Menenti@tudelft.nl; 4 State Key Laboratory of Remote Sensing Science, Institute of Remote Sensing and Digital Earth, Chinese Academy of Science, Beijing 100101, China

**Keywords:** crop water requirements, irrigation water requirements, crop coefficient, web-GIS, earth observation, evapotranspiration

## Abstract

The experiences gathered during the past 30 years support the operational use of irrigation scheduling based on frequent multi-spectral image data. Currently, the operational use of dense time series of multispectral imagery at high spatial resolution makes monitoring of crop biophysical parameters feasible, capturing crop water use across the growing season, with suitable temporal and spatial resolutions. These achievements, and the availability of accurate forecasting of meteorological data, allow for precise predictions of crop water requirements with unprecedented spatial resolution. This information is greatly appreciated by the end users, i.e., professional farmers or decision-makers, and can be provided in an easy-to-use manner and in near-real-time by using the improvements achieved in web-GIS methodologies (Geographic Information Systems based on web technologies). This paper reviews the most operational and explored methods based on optical remote sensing for the assessment of crop water requirements, identifying strengths and weaknesses and proposing alternatives to advance towards full operational application of this methodology. In addition, we provide a general overview of the tools, which facilitates co-creation and collaboration with stakeholders, paying special attention to these approaches based on web-GIS tools.

## 1. Introduction

Pressure on water use is globally increasing, and water demand for agriculture is the main driver for this pressure in many countries. The current demand of fresh water for agriculture in the world is unsustainable as recognized by Food and Agricultural Organization of the United Nations (FAO) [1]. However, in spite of these increasing pressures, irrigation intensification is required to be increased for food production for a growing population [2]. One of the possible ways to solve this dilemma could be the improvement of the efficiency in water use for irrigation to achieve a sustainable intensification of irrigated agriculture, in line with the definition of Garnett et al. [3] as “to produce more outputs with a more efficient use of all inputs (including knowledge and know-how) on a durable basis”.

In the scheme of crop management, a good first step towards the improvement of water use efficiency is the adequacy of the water applied to the actual crop requirements, pointing to the necessity of adequate estimates of the net irrigation water requirements (NIWR). NIWR is the water that must be supplied by irrigation to satisfy evapotranspiration, leaching and miscellaneous water supply that is not provided by water stored in the soil and precipitation that enters the soil [4]. Therefore, calculation of NIWR requires estimation of crop water requirements (CWR) and soil water balance where crop evapotranspiration (ET) is the main component. A huge body of knowledge has been growing in recent decades to estimate ET, CWR and NIWR. Manuals used worldwide to determine CWR and NIWR, like FAO-24 [5] and FAO-56 [6], are milestones tracking this path, closely related to those that describe the relationships between yield and water, like FAO-33 [7] and FAO-66 [8]. Nevertheless, the complex interactions between root zone soil moisture flow, salinity build up, dry-matter production, water quality degradation and opportunities to recycle water according to prevailing geo-hydrology and drainage conditions will require the use of more complex models describing the system with sufficient detail [9].

Most extension and irrigation advisory services at local and national scales were built on the wave of the “Green Revolution” to help farmers supply the right amount of water to the crops to improve the efficiency in the water use for irrigation. Nevertheless, better matching temporal and spatial water supply to the actual crop demand is a challenging key issue for sustainable intensification, in addition to nutrient supply and other agrochemical inputs. Despite its relevance and the efforts already achieved, water management still faces a development bottleneck: it requires precise information about the soil and plant conditions consistent across farms and from year to year. In addition, this information must be available at the right temporal and spatial scales that match rapidly-evolving capabilities to vary cultural procedures, irrigation and agrochemical inputs [10].

Remote sensing imagery from cameras on board satellites, aerial platforms, airplanes or similar systems has been recognized as an exceptional tool to produce spatial information about ET. Nevertheless, the lack of availability of timely images at the required spatial resolution, to be able to capture the within-field variability of crop conditions over the growing cycle, has been hindering the use of remote sensing approaches in practical applications. In 1984, in a seminar essay on the potential use of remote sensing for making day-to-day farm management decisions, Ray Jackson [11] stressed the overall importance to the growers of (a) timeliness, (b) frequency and (c) spatial resolution of data. His observations have remained relevant; but the advances in communication technology and computing, together with a large change in the data policy by National Aeronautics and Space Administration (NASA) by the U.S. government, giving open and free access via the Internet to the georeferenced Landsat images in near real time, are removing these barriers. The adoption by the European Space Agency (ESA) of the same data policy, giving free and open access via the Internet to the 10-m imagery acquired by Sentinel-2, is revolutionizing the satellite-based remote sensing system for spatial resolutions in the range of 10–30 m. In addition, an increasing number of commercial sensors at very high spatial resolution of 1–5 m, WorldView2, PLEIADES, DMC and DEIMOS, is ready to provide frequent land observations with increasing capabilities.

Currently, the operational use of dense time series of remote sensing (RS)-based multispectral imagery at high spatial resolution is able to monitor the crop biophysical parameters related with crop ET and crop water use across the growing season, with suitable temporal and spatial resolutions. One most prominent and direct application of these approaches in agriculture is irrigation management. As described by Allen et al. [12], the benefits of these methodologies with respect to most classical information sources (field measurements or general knowledge) are the possibility to cover large areas, enabling sampling at high spatial resolutions and the zonation and/or integration over diverse areas. In addition, these procedures are generally more economical than point measurements. The literature is abundant in RS-based ET models or model variants and validations of these models in different environments, surfaces and managements. Every model has strong scientific bases and is well calibrated for ET assessment at particular temporal and spatial scales. The experiences carried out within the PLEIADES project have confirmed that RS is a mature technology ready to be transferred to operational applications in irrigation management [13], and the technological transfer has already begun, where farmers find economic incentives to increase the irrigation efficiency [14]. Nevertheless, the translation of ET estimates into irrigation requirements and recommendation needs further development, and it involves additional engineering methods and operative issues. In addition, the physical meaning of the results is different for the various ET models, and these results have different applications in agriculture. Both aspects must be considered prior to recommending the most adequate model for different purposes, and from our point of view, the scientific literature is scarce in reviews analyzing these aspects.

In this framework of research and practical application, this paper reviews the basis of the most common methods based on RS for ET assessment with the focus on irrigation assessment in agriculture. We provide a comprehensive review of the basis of these models and their applicability, identifying the strengths and weaknesses and proposing alternatives to advance towards full operational application. Considering that these approaches are eminently applied, this paper also contains guidelines needed to provide a realistic estimation of remote sensing-based CWR and NIWR in operative schemes and an extensive description of the most operational decision support systems based on these methodologies.

## 2. Remote Sensing-Based Estimates of Evapotranspiration

Most of the methodologies for ET assessment based on RS data are based on the big leaf area model [15,16] and further developments of the Penman–Monteith equation. This schematization relies on the surface energy balance and the resistances approach for describing the transport of water vapor, distinguishing between bulk surface and aerodynamic resistances [16]. The Penman–Monteith equation can be applied to estimate ET once surface and aerodynamic resistances are properly determined for a crop cover of given characteristics, namely hemispherical surface albedo, Leaf Area Index (LAI) and height, as well as meteorological conditions and soil water status. In the context of irrigation scheduling, the Penman–Monteith equation has been implemented in the standard procedure for estimating crop water requirements, commonly known as the FAO-56 method [6]. In this procedure, the concept of ET under standard conditions is formalized, i.e., “from disease-free, well-fertilized crops, grown in large fields, under optimum soil water conditions and achieving full production under the given climatic conditions” This definition allows for considering a minimum value of the stomatal resistance driving the transpiration process, which essentially becomes a function of the crop development, through the above-mentioned characteristics. This approach, defined in [6] as the “direct calculation”, needs crop characteristics measured or estimated for each crop patch. Diversely, for a water-stressed crop, the surface resistance increases according to the physiological response mechanisms, which are characteristics of each species. The ET under non-standard conditions hence requires additional data to solve the surface energy balance or to compute the soil water balance.

**Figure 1:**
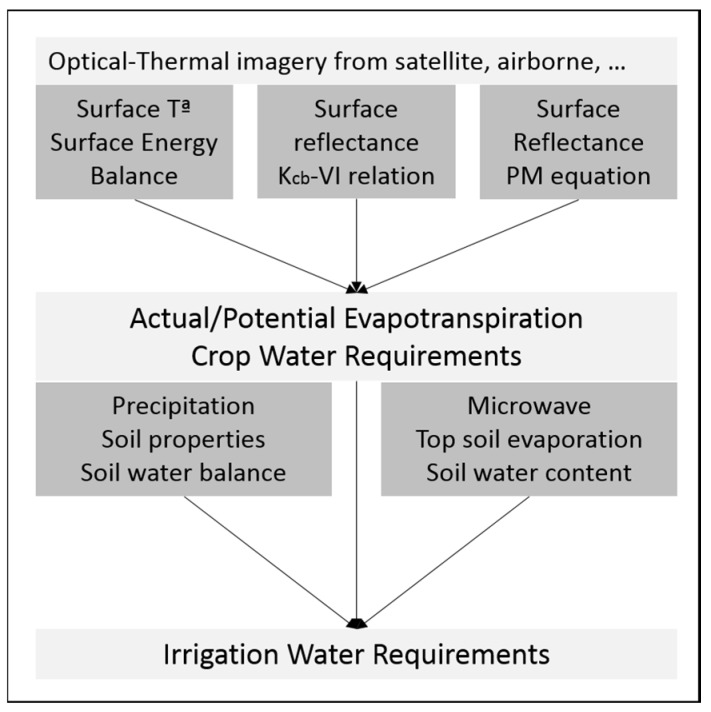
Overview of the remote sensing-based approaches for estimates of evapotranspiration and net irrigation water requirements. The spatial scale of these approaches is related to the pixel size of the utilized image data.

The direct calculation has been used to improve the definition of reference ET, ETo, by considering a well-watered hypothetical grass surface having fixed crop height (0.12 m), albedo (0.23) and LAI (2.88). Then, FAO-56 promotes the concept of crop coefficients in the so-called “two-step” procedure [5,6,17], which is now widely used in irrigation practice. In this procedure, ET is estimated as a product of two factors [6]. The first factor is the evaporative power of the atmosphere, ETo. The second factor in the “two-step” approach is the crop coefficient, Kc, which includes three parameters: a transpiration coefficient or basal crop coefficient, Kcb, the evaporative component of the bare soil fraction, Ke [18], and the water stress coefficient, Ks, which is related to the soil water content through the water balance in the root soil layer. In this framework, the Kcb is defined as the ratio between plant transpiration in the absence of water stress and reference ETo. In contrast with the strong temporal variability of ET values, the evolution of Kcb over time can be represented by a smooth continuous function. Depending on the variable measured from satellites, three main RS approaches for ET estimation have been applied: (a) based on surface energy balance (RSEB), (b) reflectance-based crop coefficient (reflectance-based Kcb) and (c) by directly applying remote sensing-based parameters into the Penman–Monteith equation (RS-PM). Figure 1 shows a schematic representation of the framework for the integration of the different models for the assessment of CWR and NIWR.

### 2.1. The Reflectance-Based Basal Crop Coefficient (Kcb)

**Figure 2:**
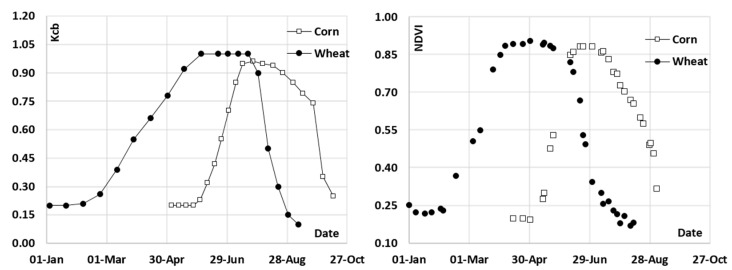
Comparison of the Kcb curves described by Wright, J.L. in 1983 [18] for wheat and corn and the temporal evolution of NDVI for both crops in Albacete (Spain) during the 2016 growing season.

The initial research relating crop development and canopy reflectance was developed during the 1970s [19,20], and much of this work had its foundation in research developed during the 1960s, as compiled by Pinter et al. [21]. Some of these authors already postulated the possible use of these relationships for the estimation of crop transpiration and the desirable use of these approaches for irrigation assessment in operative scenarios [22]. Following the development of the “two-step” procedure, some pioneers provided empirical evidence about the direct relationship between the Kcb values with the VI derived from multispectral satellite images [23,24,25] (see Figure 2).

Despite the empirical evidence, the physics underlying the Kcb-VI relationships was controversial. The arguments in favor of the causal Kcb-VI relationship include the direct relationship between Kcb and the fraction of photosynthetic active radiation absorbed by the canopy (fPAR) and the relationship of these parameters with the VIs. Some analytical approaches relating Kcb-VI and fPAR were proposed by several authors during the following decades [26,27,28,29,30,31].

The initial relationships already presented were developed in terms of empirical values of VI and tabulated or common values for herbaceous crops, such as wheat and corn. The development and popularization of different methods for the measurement of crop ET, such as lysimeters, eddy covariance and Bowen ratio techniques, provided a new source of data for the development of empirical Kcb-VI relationships, and a large number of crops were added to the classical species. Some examples are the Kcb-VI relationships derived and evaluated for potato [32], cotton [33], sugar beets [34] and vegetable crops, including garlic, bell pepper, broccoli and lettuce [35]. The advantage of using Kcb-VI is recognized for almost every crop, but the benefit of these methods applied to fruit trees is of paramount importance. The differences in local practices (planting densities, plant architecture and the management of the crop understory) have a great effect on the actual value of the crop coefficient, and studies have demonstrated the capability of the Kcb derived from VI to capture these variations. Along this line, successful developments have been made for pecan trees [36], vineyards [37,38] and apples [39], and several attempts have been made in natural vegetation [40,41].

In addition to the previous research, based on ground-based measurements of ET, special attention should be paid to those relationships based on VI data and ET estimated based on thermal-based remote sensing models [42,43,44]. These methods allow for a determination of latent heat fluxes, hence the actual ET of crops. When these methods are applied over irrigated areas (where in most cases ET can be considered under standard conditions), they result in a massive calibration of the single Kc-VIs relationships without the necessity of cumbersome and expensive field campaigns measuring ET.

### 2.2. Remote Sensing-Based Penman–Monteith Direct Approaches

As mentioned before, the direct calculation of the Penman–Monteith equation can be used to estimate the maximum fluxes of evaporation from soil (*E*) and transpiration from plant leaves (*T*) once provided with the canopy parameters related to the surface properties [45]; essentially the surface and canopy resistances (r_s_ and r_c_, respectively) and the net radiation (Rn). These parameters are, in turn, related to three parameters derived from RS data: namely, the Leaf Area Index (LAI), the crop height (*h*_c_) and the surface albedo (r). The variable r_c_ is inversely related to the active LAI and, in turn, dependent on the maximum resistance of a single leaf. The active LAI is the index of the leaf area that actively contributes to the surface heat and vapor transfer [6]. It is generally the upper, sunlit portion of a dense canopy and can be approximated by 0.5 × LAI [45]. The maximum resistance of a single leaf is crop-specific and differs among crop varieties and crop management [6], but a fixed value of 100 m/s can be considered in operative approaches [45]. The canopy architecture parameter used in the estimation of r_c_ is the canopy height. Although the formulation can vary depending on meteorological conditions (stability), it is generally accepted that, in agricultural fields under well-watered conditions, the stability correction is not needed. In addition, in most cases for irrigated environments, the radiative component of the Penman–Monteith equation is dominant over the aerodynamic term; hence, a fixed value of crop height can be considered (i.e., 0.4 m for herbaceous crops, 1.2 m for tree crops) without significantly affecting the final accuracy. There is substantial literature on the estimation of the two most relevant canopy parameters, surface albedo and LAI from VIS-NIR observations, based either on empirical relationships with different VIs or physically-based methods, such as radiative transfer models [46,47]. This approach offers the advantage of a validation based on the estimated accuracy of albedo and LAI, the latter easily measurable in the field by means of portable optical analyzers. A similar methodology is the base of the MOD16 global ET product [48,49] and further applications in natural vegetation and regional scales [50,51,52,53]. This method has been evaluated for ET estimates and irrigation management at the scale of irrigation schemes [54], in fruit trees [55,56,57] and is the basis of an irrigation advisory service operational in Italy, Austria and Australia [58].

### 2.3. The Remote Sensing Surface Energy Balance

The remote sensing surface energy balance approaches (RSEB) derive surface fluxes from the energy balance equation [59,60,61,62] by calculating the required variables from RS primary and secondary observables [63]. In particular, latent heat flux, λET, is estimated as the residual term of the surface energy balance equation:

λET = Rn − G − H
(1)

where λ indicates the latent heat of the vaporization of water (J·kg^−1^), Rn is the net radiation flux (W·m^−2^), G is the soil heat flux and H is the sensible heat flux. The main observables are the bi-hemispherical surface reflectance, which determines Rn, and the radiometric surface temperature (T_R_), derived from thermal band imagery, and used to compute the sensible heat flux. The different schemes of RSEB models differ as to how the difference between T_R_ and the aerodynamic temperature, T_o_, is addressed. This difference is needed to compute the sensible heat. T_R_ and T_o_ are clearly related [64], but this relationship is highly complex, since T_R_ depends on the temperature of the different elements that occupy the radiometer view, while T_o_ depends on surface aerodynamic roughness, wind speed and the coupling of soil and canopy elements to the atmosphere.

The simplest RS-based SEB approaches use empirical/semi-empirical methods for adjusting T_R_ to T_o_, tuned to account for the spatial variability in the roughness lengths for heat and momentum transport [65,66,67,68]. Other approaches avoid the problem by computing the aerodynamic to air gradient, T_A_-T_o_, needed to compute the latent heat flux. These methods are based on selecting pixels in the satellite image representing the extreme heat and water exchanging surfaces. Then, they calculate the spatially-distributed sensible heat flux, assuming a linear relationship between T_R_ and the near-surface air temperature gradient across the image [59,69,70,71]. Other T_R_-based approaches model the effects of partial vegetation cover on T_o_ using two-source model parameterizations [64,72], which partition surface fluxes between the soil and canopy components of the scene. This more physically-based approach does not require in situ calibration, although most implementations do require accurate radiometric temperature measurements. Anderson et al. [73] proposed an improvement of a two-source scheme by incorporating a simple description of planetary boundary layer dynamics. The resulting atmosphere-land exchange inverse (ALEXI) and an associated flux disaggregation technique (DisALEXI) are a multi-sensor thermal approach to ET mapping that reduces the need for ancillary data input and is able to deal with errors in T_R_ remote estimation by using the rate of change in T_R_ observations [74,75].

The partitioning of available energy through T_R_ inherently accounts for the increase of plant temperature under water stress conditions [22,76], and successful model validation under water stress conditions has been regularly published [62,77,78]. A comparison between a two-source model and an internally-calibrated model over herbaceous crops [79] showed a reasonable agreement with ground measurements. This approach is very attractive in the calibration and validation of the other approaches presented here [80] and for applications such as water stress assessment. Water stress is an important indicator for the evaluation of adequate crop water management in precision agriculture. Stress indicators are useful to diagnose the causes of crop yield variability and develop management strategies [81] in water-limited environments. The most classical indicator of crop water stress that uses RS data without using direct measurements [63] is the crop water stress index (CWSI) based on the difference between air and canopy temperature [22,76]. Later development of the CWI considered the effect of partial canopy covers in the surface temperature, as is the case of the surface-air temperature and VI relationships [62], and further developments and simplifications [60,82]. The literature is profuse in the use of CWSI or similar indicators for the assessment of crop water status and irrigation scheduling [83,84,85,86]. These indices and other diagnostic tools are indicators of the plant water status, revealing the effects of the water deficit, but they cannot predict the irrigation timing or amount needed to maintain the crop under optimum conditions. Other approaches to water stress, such as the hyperspectral indices, have gathered promising results in agriculture [87,88,89,90] in addition to other stress indicators based on multispectral satellite signal, such as the Normalized Difference Water Index (NDWI) [91], and are attractive for extensive applications in natural vegetation.

### 2.4. Coupling Models

The soil water balance models based on remote sensing data (RS-SWB models) provide continuous and predictive estimation of the soil water content, cumulative ET [92] and irrigation requirements. However, for an adequate estimation of these components, the SWB model requires knowledge of the water inputs, precipitation and irrigation, and the soil hydraulic properties, i.e., actual and maximum amount of soil water storage in the root zone, if classical static volume balance approaches are used. Accurate values of maximum and actual water content are necessary in every SWB model, although both concepts could be represented with different notations [6,17,73,93]. The actual content can be estimated when the balance is maintained for long periods, departing from dates when the soil can be considered at full capacity, but the uncertainties about the spatial variability of the water inputs (mainly precipitation) and the inaccuracy in estimating other components result in significant bias at large spatial scales and for long periods. In addition, the practical operation of these models is also limited by the narrow knowledge about the soil properties, which define the water retention, field capacity and wilting point, in addition to the actual root depths for most of the crops growing in heterogeneous areas.

Within the six approaches classified by Wang-Erlandsson et al. [94], for the estimation of root zone water storage capacity, RS-based studies are generally based on field observations and look up tables [95,96,97,98]. Nevertheless, some recent studies propose the optimization-calibration and inverse modelling approaches with diverse purposes. Some approaches assimilate into the soil water balance models, either water stress estimates based on canopy temperature [99,100] or ET estimates based on SEB models [73,92,95,101], in order to calibrate the fraction of water depleted derived from the water balance model. In a slightly different approach, some authors propose the integration of actual ET values in order to calibrate the soil water balance model in terms of the root zone storage capacity [94,102,103,104]. The rationale of these approaches is that any empirical approach to the plant water stress, or alternative formulations as those based on the canopy temperature, must be equivalent to the soil water stress, a stress index based on the parametrization of the soil properties [101]. Both approaches to water stress result in similar values only if the SWB model is properly initialized and maintained. Therefore, those variables with large uncertainties, as is the case of the fraction of water depleted or the root zone storage capacity, can be calibrated.

However, the lack of information about the actual irrigation scheduling adopted by the farmers is the critical limitation when applying soil water balance models. Irrigation criteria adopted by farmers depend on several factors related to the operation and management of irrigation conveyance and distribution systems and to farmers’ perception about the best time and duration of irrigation applications. This issue might be addressed by using deterministic or stochastic approaches to parametrize farmers’ behavior [105]. Still, remote sensing is very valuable in this context since the knowledge of the actual development of crops is one of the most important variables in the description of this process.

### 2.5. Advances Achieved in Proximal Remote Sensing

The denomination of proximal remote sensing includes a wide range of devices mounted in ground-based and aerial platforms, including aircraft and unmanned aerial vehicles (UAV). In recent years, we have assisted the increase in the use of UAVs in agriculture. The reasons for this increase are multiple: affordable cost, relative simplicity for operation and images post-processing, in addition to exceptional technical advances in the cost reductions and the size of sensors related to the Global Positioning System, pre-programmed flights, inertial movement units and auto-pilots [106].

These systems can fill some of the gaps in our observational capabilities exclusively based on extra-terrestrial platforms. Regarding the assessment of ET and irrigation requirements, the methods analyzed in this work can be also based on the images acquired by UAVs, providing the needed spatial resolution in some agricultural areas and reducing the impact of cloudiness in the optical satellite images. Although the compatibility between satellite and proximal RS is evident, the images based on UAV platforms are mainly used in applications that require exceptional spatial resolution or when the phenomena analyzed occur in short temporal periods [107,108]. In this line, several approaches analyzed the use of very high resolution images for the assessment of nutrition or water stress indices at the scale of the row or the tree crown scale [87,88,89,90,109], gathering promising results in agriculture.

### 2.6. Strength and Weakness of the RS-Based Models for Irrigation Assessment

The great strength of the reflectance-based models from the point of view of crop irrigation management is the capability to estimate the potential crop transpiration, based on the temporal evolution of the RS-based Kcb and the actual ETo values. This ability of VI enables the description of the photosynthetic magnitude of the canopy [110,111,112]. Reflectance-based basal crop coefficients represent the “potential” or maximum ratio between transpiration and ETo for the canopy, as happens for an unstressed canopy following the definition of the Kcb concept. The advantages of VI-based Kcb estimation for irrigation assessment are clear as proposed by Allen et al. [12] in a review of the methods used for ET estimation: (a) probably the simplest method to introduce RS data is the operational application of the Penman–Monteith formulation for ET assessment known as the “two-step” methodology, which enables quick analyses that can be made by mid-level technicians; (b) large areas can be covered; and (c) a very high spatial resolution if aerial imagery is used. As indicated by the same authors, the main weaknesses of the methods based on the Kcb-VIs for crop ET assessment are: (a) the estimation of the evaporation component (from soil) is less certain than the transpiration component because of the lack of a direct relationship with vegetation amount; (b) the relationships tend to overestimate transpiration under conditions of water shortage; and (c) the relationships may vary with the type of vegetation; stomatal control (and thus Kcb-VI relationships) can vary among types of vegetation.

The variation in the Kcb-VI relationships can be perceived in the compilation of equations based on the most used multispectral vegetation indices, as is the case of the normalized difference vegetation index (NDVI) and the soil adjusted vegetation index (SAVI), presented in Table 1. The relationships shown in Table 1 reveal a similarity for those relationships that reach the maximum NDVI or SAVI values, typically around 0.9 for NDVI and 0.7 for SAVI, resulting in a mean Kcb value of 1.14 and a standard deviation (SD) equal to 0.08. The main differences appear for bare soil, the corresponding NDVI value being around 0.15 and the SAVI value around 0.1. Some relationships consider a minimum Kcb equal to zero for bare soil [30,31,34,113], arguing that no transpiration occurs for bare soil conditions. Other Kcb-VI relationships are established in terms of Kcb values greater than zero for bare soil conditions [23,35,37,114]. This has been recurrently analyzed in the literature, as early as Wright [18] and Allen et al. [6]. Torres and Calera [115] demonstrated empirically that this residual Kcb can be expected for bare soil conditions and should be attributed to the evaporation component rather than plant transpiration [113]. The discussion about the most adequate minimum Kcb in reproducing the crop ET is still open, and further detailed analysis will be necessary for providing a practical solution.

Differences in the VIs measured with different instruments, and the difficulty to measure canopy transpiration, in addition to the effects of the crop physiology and structure in the ET process could be the basis of the mentioned discrepancies. The effect of the measurement instruments depends on the sensor’s spectral and radiometric resolutions [116], differences in the acquisition angle [117,118], atmospheric correction, sensor degradation and the correctness of the calibration process [119]. These sources of uncertainty can be minimized by applying cross-calibration approaches and ensuring the compatibility of the data-sources [116]. Additional differences might be attributed to the well-documented variances in the stomatal response for the different species [120,121] in contrast to the insensibility to these changes of the VI used for the assessment of Kcb.

Some Kcb-VI relationships exhibit very good agreement for different crops. Odi-Lara et al. [39] and Campos et al. [122] found that the relationship described by Campos et al. [37] in row vineyards was adequate for ET assessment in apple trees and Mediterranean holm oak savanna. Hornbuckle [123] concluded that several relationships, developed for multiple different crops [124], are valid for the assessment of vineyard ET in Australia. Melton et al. [125] proposed the use of a generalized relationship for real-time and operational purposes and apply crop-specific relationships a posteriori, when information about crop architecture is available.

The RS-PM methods are also in surface reflectance, thus the strength and weakness are similar to the reflectance-based Kcb models. The RS-PM approach solves the problem of the estimation of the resistances in the Penman–Monteith formulation for the conditions of a well-watered canopy. The parameters used in the respective solutions are strongly related with RS data and the key parameters, LAI, albedo and hc, and these variables describe smooth-continuous functions that can be easily interpolated over time. The weaknesses are in the crop-specific LAI-Vis’ and hc-Vis’ relationships, the impossibility to reflect the effect of the water stress in the ET process and the role of the soil evaporation. In a complete analysis of the LAI-VIs, Anderson et al. [128] concluded that the LAI-Vis’ relationships were relatively stable for two different crops (corn and soybean) using determinate VIs. Similarly, Vuolo et al. [129] concluded that the models and calibration parameters used to estimate LAI from VIs can be transferred across different environments, management practices and for multiple crops, including alfalfa, corn, sugar beet and vineyards. In addition, according to the sensitivity analysis published by Consoli et al. [55] and D’Urso [45], the deviation of ET values by considering a constant value of hc, over a wide range of leaf area indices, is lower than 10%. Furthermore, the availability of sensors with improved spectral and spatial resolution, such as MSI on board the Sentinel-2 satellite, allows the application of inversion methods to canopy radiation transfer models to estimate crop biophysical parameters with greater reliability compared to other methods. These methods take into account the bidirectional reflectance distribution effects of the canopy, as well as the actual illumination-viewing geometry of the sensors. Artificial neural networks have proven to be effective in terms of accuracy and computational time [130], and tools are provided in freely available packages, such as the Sentinel Application Platform (SNAP), developed by the ESA to estimate LAI, fractional vegetation cover, and other parameters from Sentinel-2 data (https://sentinel.esa.int/web/sentinel/toolboxes/sentinel-2). Experimental studies have shown the accuracy of this approach for LAI or ET estimation in different environments and crops.

The weakness of RSEB approaches is the representativeness of the ET estimates over time because they provide an instantaneous estimation of the ET at the image acquisition time. This instantaneous value must be extrapolated to daily amounts on a physical basis, such as the conservation of the energy partitioning [79] or the stability of the crop coefficient [69]. The time gaps between estimates of ET for all satellite systems may bias daily-to-seasonal estimates. As pointed out by Allen et al. [69], the effects of precipitation or irrigation events occurring between satellite overpasses may be missed, resulting in underestimation of seasonal ET. In addition, processing of images impacted from recent precipitation events could lead to an overestimation of the seasonal values of ET if these images are used in the interpolation. In the framework of NIWR estimates, another operative issue is the adequate interpretation of ET data obtained under water stress conditions. According to the definitions provided in the Introduction, NIWR is the amount of water that should be applied in order to maintain the crop transpiring at its potential rate. Acquiring ET data under water stress conditions could lead to an underestimation of NIWR if the actual values are not compared with the potential (and eventually desired) ET rates for the analyzed canopies. In addition to these weaknesses, the limited availability of thermal observations in terms of spatial and temporal resolution hampers the development of operational applications of surface energy balance from remote sensing.

In general terms, the main difference between RSEB models with respect to RS-PM and reflectance-based Kcb approaches is the assessment of water stress, but the three approaches should result in similar values when applied under non-water limited conditions. Singh and Irmak [44] found that a Kc-NDVI relationship derived from the SEBAL model (Surface Energy Balance Algorithm for Land) is able to reproduce the actual ET measured with a Bowen ratio station. Tasumi et al. [42] concluded that the ET estimates from a Kc-NDVI relationships correspond well with the results of the model METRIC (Mapping Evapotranspiration aT high Resolution with Internal Calibration) applied for multiple crops in an irrigated area in Idaho. Rafn et al. [43] demonstrated that the results of three Kc-NDVI relationships, derived from empirical or analytical approaches, are within the range of ±10% of the ET estimate based on the METRIC model. Hunsaker et al. [33] found similar yield and water productivity in cotton plots irrigated following the Kc-NDVI relationship and the Kc values recommended in the FAO-56 manual (adapted to the area, planting dates and crop development). Rubio et al. [80] published a direct comparison of two RSEB models, the RS-PM approach and the reflectance-based Kcb. These authors concluded that the RS-PM and reflectance-based Kcb models are in agreement with each other, although these authors did avoid the direct comparison of both approaches with RSEB models because of their different nature. Similarly, D´Urso et al. [13] obtained a comparable accuracy when the reflectance-based Kcb and the RS-PM models are applied to herbaceous crops, like corn, alfalfa and wheat. Gonzalez-Dugo et al. [79] compared three RSEB models and the reflectance-based Kcb approach for the assessment of ET in irrigated herbaceous crops. These authors obtained similar accuracy for every model, but the two-source RSEB and the reflectance-based Kcb were the approaches with the lower RMSE. In the view of the results, we can conclude that all models provide similar results in the assessment of the ET of irrigated herbaceous crops (homogeneous canopies under non-water-limited conditions). Further studies should analyze if these differences in the accuracy of the model have a measurable impact in irrigation assessment systems.

Although each model has been evaluated in other crops, as is the case of horticultural and fruit trees, we did not find comparative studies running different models on the same conditions. Future studies comparing different approaches for these crops and in operative schemes will provide further insights on model performance. An interesting research line, no yet translated to the scientific literature, is the implementation of the models in the daily routine in operational scenarios. The development of this research line, quantifying the actual improvements in terms of water productivity or economic returns, is necessary in the short term and will provide arguments for the adoption of these technologies in the sector. In addition, the accumulation of knowledge and experimental evidence will provide certainty about the actual consequences of the propagation of the errors associated with the models used for the ET assessment.

In addition, the interest of these methods goes beyond the perspective of irrigation management. Although it is not discussed in this paper, the output of this remote sensing-based soil water balance paves the way for water accounting at the pixel scale for water governance and environmental purposes. The methodologies discussed here can be used for the assessment of irrigation performance indicators in large areas [131,132] and the analysis of the sustainability of irrigated systems [133]. Special mention deserves the analysis of water productivity in great areas [134] or at the global scale as proposed in the FAO-WAPOR program (FAO WAter PROductivity, available at http://www.fao.org/in-action/remote-sensing-for-water-productivity/en/). Finally, we identify promising perspectives for the use of this methodology together with on-site flowmeters to enforce legal rules about monitoring permitted abstraction volumes to use for irrigation [135].

## 3. Operational Use of Remote Sensing for Irrigation Water Management

### 3.1. Monitoring the Crop Development at the Right Spatial and Temporal Scale

Monitoring crop development and crop ET over the growing season for the purpose of irrigation management requires dense time series of multispectral imagery at a spatial resolution high enough to resolve within-field variability and delivered in real time. The spatial and temporal resolution of the resulting maps of ET and NIWR depend on the pixel size of the input imagery. In addition, and given that the crop evolves rapidly in most cases, single satellite sensors or platforms cannot adequately capture these changes due to their limited temporal resolution and the impact of cloudiness in the optical and thermal satellite images. In a commentary about the future of the remote sensing-based ET, Fisher et al. [136] highlighted that neither planned nor existing space missions have been specified to fully meet the spatial, temporal, spectral and accuracy requirements outlined for complete ET-based science and applications. However, virtual constellations of planned and existing satellites help to overcome this limitation by combining all available observations to mitigate the limitations of any one particular sensor [137]. For models based on reflectance-based VI and further secondary variables, which rely on VIS-NIR imagery, the pixel size ranges usually between 5 and 30 m using most of the commercial (World View, Rapid Eye, DMC and DEIMOS) and free images from the sensors on Landsat 8 and Sentinel-2a currently in orbit. Accordingly, the virtual constellation of Landsat 8 and Sentinel-2a currently provides, at no cost, a time resolution of around one image per week, which can be considered as a minimum for the adequate monitoring of crop development. The time series of both sensors are accessible through the USGS (http://glovis.usgs.gov/) and Copernicus (https://cophub.copernicus.eu/) sites. In addition, some companies, like Amazon S3 (https://aws.amazon.com/es/public-data-sets/landsat/) and Google Earth Engine (https://earthengine.google.com/), are offering catalogs of satellite imagery from both sensors at the planetary scale, as well as additional cloud computing capabilities. The use of multi-sensor virtual constellations is the only way to ensure the frequent availability of cloud-free images. Yet, the actual number of images effectively usable in an area or period can be seriously impacted by clouds, even considering multiple platforms. Some initiatives, like the recent launching of Sentinel-2b, foreseen for 2017 (https://earth.esa.int/web/guest/missions/esa-operational-eo-missions/sentinel-2), will increase the availability of cloud-free imagery. Currently, as presented in the next section, the demand for irrigation recommendations and the implementation of operational services is primarily in arid and semiarid areas characterized mostly by low precipitation and high atmospheric demand, which are only minimally affected by clouds. However, the implementation of these methods in areas of significant cloudiness must also be considered.

The availability of dense time series of images at the global scale also implies the necessity of massive storage, automatic download and archiving and computing capabilities as provided by the companies cited above. However, the accessibility to the images (free of charge and near-real-time processing capabilities) provided by the ESA and NASA incentivizes the development of the operative services analyzed in this paper and opens the possibility of the massive use and validation of the cited approaches. In this line, we recognize the effort made to make publicly available the codes of SEBS (Surface Energy Balance System, http://pcraster.geo.uu.nl/projects/applications/sebs/) or METRIC (https://cran.r-project.org/).

ET products based on RSEB can have medium spatial resolution for most operational satellites. The pixel size ranges from 100 m for the thermal sensor on board Landsat 8 to 1000 m for MODIS-AQUA, MODIS-TERRA and Sentinel-3; additional data sources and downscaling algorithms and interpolation methods can be used to improve the temporal and spatial resolution. From the point of view of crop management, the strength of these models is the assessment of surface ET also under water stress conditions and further indicators of water stress and irrigation performance. Nevertheless, the spatial resolution of thermal images provided by the most operational platforms is not appropriate for small agricultural fields [12] since the pixels may overlay broad mixtures and densities of crops so that surface temperature signals are mixed and the ET retrievals are difficult to interpret. Therefore, from an operative point of view for irrigation management, the procedures based on satellite canopy temperatures seems to be complementary with that previously described, providing an independent quality control in the suitable areas. Efforts are ongoing to implement disaggregation techniques to increase the effective spatial resolution from satellite thermal imagery, reaching spatial resolutions comparable to the most common multispectral images [138]. In addition, the spatial resolution can be improved up to 2–5 m from aerial images, and growing advances on the use of airborne thermal cameras show very promising perspectives to produce temperature maps at very high spatial resolution [88,139].

### 3.2. RS-Based Irrigation Scheduling: Implementation

As presented and discussed in the previous sections, time series of current multispectral imagery that provides canopy reflectance can be directly converted, either through Kcb-VI relationships, or using more complex models, into maps of Kcb, or related variables describing the potential crop water use. Gap filling techniques between images close in time allow the production of daily maps of the variables of interest, LAI, h_c_ or, directly, Kcb, taking advantage of smooth-continuous curves described by these parameters and so avoiding the pernicious effects of cloudiness. The product of these Kcb maps and reference evapotranspiration, or the solution of the PM equation using RS inputs, directly provides the daily potential transpiration on a pixel by pixel basis. For the adequate determination of NIWR, both VI-based Kcb RS-PM models require the assessment of soil water content. These approaches estimate crop ET on the noted models, and this ET is connected to the water balance in order to update the water depletion in the soil layer accessed by the roots. Irrigation timings and amount assessment will depend on the estimates of water depleted and water holding capacity in the root zone. For these reasons, some of these approaches have been integrated into a classical soil water balance, like that described in FAO-56 [6], demonstrating good performance for the assessment of irrigation water requirements [96,140,141] in comparison with actual irrigation data. The literature is replete in soil water balance models, with different degrees of realism and complexity, but the approaches based on remote sensing data are generally based on relatively simple models [95] because these approaches have a clear inclination for operational applications at large scales. For these scales, detailed information about the soil properties is scarce [73]. According to the FAO-56 procedures, it is possible to calculate these RS-based NIWR irrigation water requirements also under water stress, as is used either in controlled deficit irrigation or in supplemental irrigation. Knowledge of the desired water stress degree is required, and further calibration of the methodology and the evaluation of irrigation management using diagnostic tools is always recommended.

In these models, soil evaporation is calculated by separately applying a soil water balance in the soil top layer as proposed by Allen et al. [6] and Torres and Calera [115]. This approach requires the knowledge of the irrigation timing and amount, which is generally unknown for great areas. Alternatively, some authors working at large scales, with scarce field data, proposed the concept of a synthetic crop coefficient [113] that accounts for mean soil evaporation derived from canopy cover estimates. Microwave remote sensing could provide insight on the bare soil evaporation, although the scales of observation for the current sensors SMAP (Soil Moisture Active Passive) and SMOS (Soil Moisture Ocean Salinity) (20 km) [142,143] is too coarse for the agriculture scale of interest.

Some initiatives implementing satellite-based irrigation advisory services have been developed in Southern Italy, with IRRISAT (Irrigation assisted by Satellite, http://www.irrisat.it), in Lower Austria, with EO4Water (Earth Observation for Water resources management, http://eo4water.com), and in Southern Australia, with IRRiEYE (South Australian Trial for a Satellite Irrigation Advisory Service, http://www.irrieye.com). These systems are based on the RS-PM method [58]. Thus, the calculation of crop ET and suggested irrigation depth (pixel and plot scale) is based on the LAI calculation from surface reflectance values and meteorological data. Remotely-sensed data from the virtual constellation of Landsat 8, Sentinel-2 and DEIMOS are used to derive crop parameters (LAI and surface albedo) on a weekly basis. Information is released to end users by using a web-GIS tool, developed in an open-source software environment and implemented in three different areas. The structure of the web-GIS has been adapted to each area considering the requirements of the local users. The IRRISAT approach has proven that economic benefits generated by such advisory services are able to fully repay the initial investments, creating advantages for the environment and opportunities for all of the users of water resources. Accordingly, IRRISAT has been deemed a “best practice” for agricultural applications by EURISY (Non-Profit-Organization aiming to promoting the benefits of Space to European Society, see http://www.eurisy.org/good-practice-campania-encouraging-the-sustainable-use-of-irrigation-water-in-the-region_85) and by the International Selection Committee of the call for “Best Sustainable Practices on Food Security” for EXPO 2015 in Milan (Italy). In the specific context of Consorzio of Sannio Alifano, Campania Region, the overall results in terms of cost-benefit analysis, obtained comparing the 2012 irrigation season (pre-IRRISAT) and 2013 (post-IRRISAT), demonstrate water savings of about 18%, while a survey on a sample of monitored farms highlights peak savings of about 25%–30% without loss of production [144].

An approach using satellite data, mobile phones and web-GIS tools for information delivery is the IrriSatSMS system (Irrigation Water Management by Satellite and SMS) developed in Australia by CSIRO (Commonwealth Scientific and Industrial Research Organisation). The system is based on the NDVI-Kcb relationship [123] and was originally applied for vineyards in the Murrumbidgee Irrigation Area, but the current geographic area covers the entire Australian continent. The IrriSatSMS system aims to simplify input data collection requirements and reduce both the costs and complexity of information output [145]. The core of the system was initially a server that acted as a data collection portal for various data feeds and a processing engine to convert these data into usable irrigation management information. The most recent version makes use of the Google Earth Engine for the image processing and algorithm implementation. Originally, the system relied mainly on a Short Messaging Service (SMS) interface to communicate with irrigators directly on their mobile phones and later development included a web-interface (https://irrisat-cloud.appspot.com/). The web interface is easily accessible; the target fields can be defined (drawn) by the user; and the information contained in the system is well presented and easily reached. Some information about the crop type, management, growing cycle and soil properties is required in order to complete the water balance.

In the framework of the NASA Terrestrial Observation and Prediction System (TOPS) [146], an application for near-real-time mapping of crop canopy conditions and associated CWR at the resolution of individual fields has been developed. The TOPS Satellite Irrigation Management Support (TOPS-SIMS) integrates satellite observations from Landsat and MODIS with ETo from meteorological information and ancillary data on crop type and site-specific conditions. The initial implementation provides a capability for mapping fractional cover, associated basal crop coefficients, and ET over 3.7 million ha of farmland in California’s Central Valley. A generalized NDVI-Kcb relationship is used for near-real-time mapping Kcb and ET. Refinements introducing crop-specific NDVI-Kcb relationships are introduced a posteriori when this knowledge is available [125]. A web-based user interface provides access to visualizations of TOPS-SIMS (https://ecocast.arc.nasa.gov/simsi/). The variable and date visualized can be selected, and the data associated with the plot analyzed can be downloaded in numerical and graphical formats.

**Figure 3:**
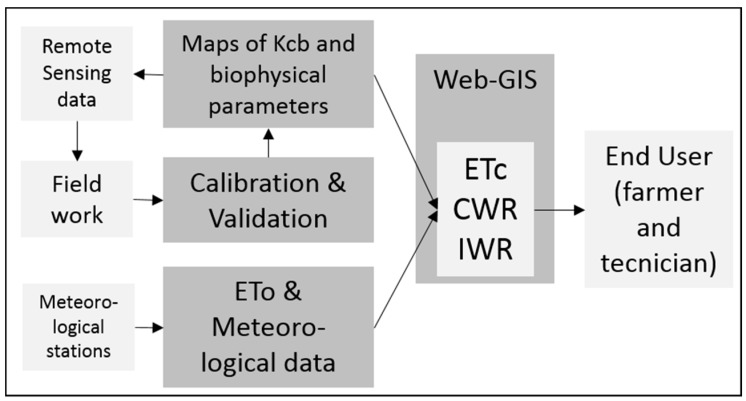
Scheme of the modular system based on the integration of remote sensing and weather observations into a web-GIS, to provide users with irrigation scheduling, matching the water supply to crop water demands. CWR, crop water requirements; IWR, irrigation water requirements.

In Southern Spain, a first experience was developed in 2005, by using time series of Landsat 5 images to obtain Kcb curves based on NDVI temporal evolution and displaying them on SPIDER (System of Participatory Information, Decision support and Expert knowledge for irrigation River basin water management, http://maps.spiderwebgis.org/webgis), a web-GIS based on open-source software developed by the University of Castilla-La Mancha. SPIDER has evolved from a 2005 prototype, and it is currently providing time series of Sentinel-2a and Landsat 8 imagery and derived products for the whole Iberian Peninsula, covering Spain and Portugal (around 600,000 km^2^). SPIDER is able to display time series of raster and vector maps, adding the capability to also display time trajectories of pixel-based values for the periods defined by the user. The main layers displayed by the systems are ETo maps, color composition RGB, NDVI, Kcb and CWR values, 24 hours after image delivery in the web-portals of Landsat 8 and Sentinel 2A by USGS and Copernicus, respectively. The image processing is off-line, and a normalization process allows the operation of multiple image sources as a multi-sensor virtual constellation; see Figure 3. A mobile app version of SPIDER web-GIS (Agrisat App) was released in 2016 and is available in the most common digital distribution platforms for mobile devices.

An additional system with interesting applications in agriculture is EEFlux (Earth Engine Evapotranspiration Flux, accessible at http://eeflux-level1.appspot.com/). EEFlux operates on the Google Earth Engine system and has been developed by the consortium of the University of Nebraska-Lincoln, Desert Research Institute and University of Idaho with funding support by Google. The system provides ET and reference ET estimates based on the METRIC model [69] applied to Landsat images around the globe. EEFlux is calibrated by assigning values to the ratio between actual ET and reference ET for the “hot” and “cold” parts (pixels or group of pixels) of the surface temperature spectrum of the scene [147]. EEFlux differs from the previous systems analyzed in the nature of the ET estimates that EEFlux provides. This system could be complementary to the previous models in the determination of the water stress, since it is based on the estimation of the actual values of ET, accounting for the water stress conditions. The automated calibration in EEFlux is still evolving, but EEFlux shows promising perspectives to reach actual ET.

### 3.3. Comparison of the Decision Support Systems Based on Web-GIS Technology

Table 2 shows the main characteristics of the web-GIS-based decision support systems analyzed in the text. The development of these operational systems for the assessment of water management confirms the maturity and the applicability of the methodologies reviewed in this paper. The advantages and improvements over traditional irrigation advisory services, based on field measurements and Kc-tabulated values, are the capability of the satellite-based system to reflect the actual conditions of the canopy, covering large areas and increasing the efficiency of field work.

The basic information provided by each system is similar: vegetation indices, color composites and core biophysical parameters derived from satellite data and related with the water use, like crop coefficients. All of the systems take into account the necessity of spatio-temporal analysis, and the user can visualize the images and query the information for different dates or time periods. An interesting option in all systems is the capability to display the location of the user or web-connected device in the maps. This geolocation, with the reference of the most recent satellite images, can be used to identify areas of interest in the field, like zones with unusual crop development. An additional point of general agreement is that weekly is the best compromise of timing for using and receiving the information about plant status and CWR.

The information provided and the calculation procedure varies between the systems analyzed. IrriSatSMS has powerful processing capabilities because it is able to calculate, on-the-fly, a soil water balance for the user-drawn polygon. The system is able to estimate and update the actual NIWR and soil water content based on the information provided by the end user. In comparison, EEFlux is able to estimate actual ET for the analyzed area, but without additional knowledge requirements. The information about NIWR and other components of the satellite-based soil water balance can be displayed in other web-GIS tools, like SPIDER or IRRISAT, but must be processed off-line. An example of the implementation of an RS-based soil water balance for the whole Iberian Peninsula at the pixel scale in irrigated areas can be seen in the SPIDER group named SPIDER-CENTER (http://maps.spiderwebgis.org/login/?custom=spider-center). This project is funded by the Spanish Ministry of Agriculture (for further information and accessibility, the reader is referred to http://www.magrama.gob.es/es/desarrollo-rural/temas/centro-nacional-tecnologia-regadios/nuevas-aplicaciones-tecnologicas/).

This difference in the processing capabilities also implies a substantial difference in the data and knowledge requirements. While SPIDER or IRRISAT can display the results of the models based on land use and soil properties maps, IrriSatSMS makes use of the knowledge of the end user in terms of soil properties, crop, planting dates and management. An additional difference between the various systems is the accessibility to pixel- or plot-based information. IRRISAT, EO4Water and IRRiEYE provide information at the pixel and plot scale. IrriSatSMS emphasizes the plot scale. SPIDER and TOP-SIMS allow the direct comparison of multiple pixels or small grids. SPIDER provides a dynamic multiple parameter chart with the temporal evolution of the selected parameters for different locations. This capability opens the possibility to show and compare the spatial distribution of the CWR or related variables and may be of interest for the analysis of crop uniformity. Although irrigation and other tasks are currently planned and performed for the whole plot, new machinery for variable rate irrigation is becoming available. The accurate generation of spatial irrigation recommendation, as is the case of NIWR maps at the pixel scale based on RS, is essential for the implementation and evaluation of variable irrigation ratio technologies [148].

Additional conclusions can be extracted if the methodology is analyzed from the perspective of the end user. The farmers willing to adopt these techniques are familiar with point soil water content sensors in such a way that they are able to check with their own knowledge the reliability of RS recommendations, comparing them with other sources of water requirement estimation. For this reason, easy access to timely information is crucial. Direct access by farmers in real time to the images in the way of the usual RGB color combination is very useful. These RGB/NDVI images enable farmers to gain confidence in identifying some details in the images that they have observed directly in their fields, such as sprinkler failure and non-uniform water distribution effects. In addition, the temporal evolution of the spectral vegetation indices or related parameters obtained during several growing seasons helps to compare the effects of management strategies (i.e., planting dates, fertilization strategies or the performance of different varieties). An interesting complement is the identification of phenological stages based on the temporal evolution of crop reflectance. Regarding our knowledge, no operational systems are providing this kind of information directly to the farmers. However, it is necessary to highlight the advances already achieved in this field [149,150] and the necessity of this information for the scheduling of agricultural tasks.

### 3.4. Predicting CWR a Week Ahead

Providing advice about CWR in operative scenarios, one week ahead seems to be a reasonable expectation, providing enough time and ensuring the accuracy of the CWR forecast. The relevance of this predictive product was already highlighted by [4] and clearly recognized by the traditional irrigation advisory services, but the remote sensing community was primarily interested in the accuracy of RS-based ET estimates. The prediction of CWR one week ahead allows for planning irrigation scheduling adapted to the power supply rates, water availability, irrigation systems, precipitation probability and farmer´s availability.

Predicting CWR one week ahead requires the extrapolation of the Kc-Kcb data and weather forecasts for ETo prediction. CWR forecast is a logical step in the reflectance-based Kcb models [35,125], although some of the operational systems, like TOPS-SIMS, still do not incorporate this product. A prediction of CWR is fully operative in IrriSatSMS and is under development for the IRRISAT, EO4Water and IRRiEYE systems based on LAI-VI relationships [151]. A commercial development of the CWR prediction based on Kcb-VI relationships has been developed in Spain (http://www.agrisat.es). The initial approach is based on a generalized Kcb-VI relationship following previous approaches discussed in this paper [123,125].

Considering that ETo can be estimated from common meteorological data, ET can be calculated from short-term numerical weather forecast. Two complementary methods with different spatial scopes and accuracy have been introduced. The first one is to use the full power of numeric weather forecast to determine the variables required to compute ETo according to the FAO-56 formulation. The second one is based in daily temperature forecasting by using it as the input into the Hargreaves and Samani equation to estimate ETo [6]. The latter method should be restricted to areas where the Hargreaves and Samani equation works well (no windy areas, no coastal areas) and where no forecasting of other meteorological variables than temperature is available. An inter-comparison analysis has been recently published considering ensemble forecast models up to five days and a spatial resolution of 7 km [152]; this study, based on COSMO-LEPS data (Limited-Area Ensemble Prediction System provided by the European Consortium for local-Scale MOdelling), has evidenced the robustness and reliability of ETo forecasts with the PM equation.

**Figure 4:**
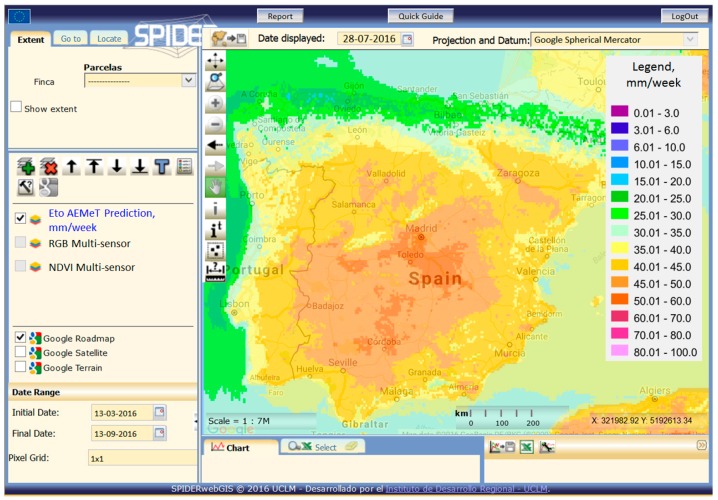
Weekly reference evapotranspiration ETo forecasting provided by Agencia Estatal de Meteorología (AEMET) displayed by the system SPIDER web-GIS.

Computing ETo according to FAO-56 from weekly numeric weather forecast is the preferred option. Maps of weekly predicted ETo are routinely provided by the Spanish Meteorological Agency, AEMET (Agencia Estatal de Meteorología). The prediction is based on the High Resolution Limited Area Model (HIRLAM) and the European Center for Medium-range Weather Forecasting (ECWMF) models. The spatial scope of this product is the Iberian Peninsula, as presented in Figure 4, and the spatial resolution of the raster map is a pixel size of 5 km. The ETo predictions are routinely compared with the weekly measured ETo maps provided by the same agency and ETo values obtained from ground stations (www.siar.es). Finally, the adequate estimation of CWR requires the extrapolation of reflectance-based Kcb, or related variables, like LAI and hc for RS-PM methods. This extrapolation takes advantage of the smooth shape of the Kcb curves derived from time trajectories of NDVI (see Figure 2). Therefore, the time trajectory of the Kcb and LAI or related parameters is suitable to be extrapolated using previous dates for short periods, as is the case of one week.

## 4. Conclusions and Perspectives

The experiences gathered during the past 30 years support the operational use of irrigation scheduling based on spectral inputs. Currently, the operational use of dense time series of multispectral imagery at high spatial resolution allows monitoring of crop biophysical parameters related with crop water use during the growing season with unprecedented temporal and spatial resolution. This information is needed and highly appreciated by the end users, such as professional farmers or decision-makers, but several steps are necessary prior to introducing this information into the day-to-day routine of irrigation farming. The information about crop water requirements must be provided with sufficient future prediction, and one week ahead seems to be a reasonable lead time. In addition, the end users require access to this information and to the time series of images in an easy-to-use way and in near real time. This information can be provided by using the improvements achieved in web-GIS methodologies and further developments, like mobile apps. The advancements in crop ET assessment, the accessibility to satellite images and the availability of accurate forecasting of meteorological data allow for precise predictions of crop water requirements.

## Figures and Tables

**Table 1:** Compilation of Kcb-VI relationships found in the literature.

Crop	Equation	Reference
Corn	Kcb = 1.36 × NDVI − 0.06	[23]
Wheat	Kcb = 1.46 × NDVI − 0.26	[30]
Cotton	Kcb = 1.49 × NDVI − 0.12	[33]
Wheat	Kcb = 1.93 × NDVI^3^ − 2.57 × NDVI^2^ + 1.63 × NDVI − 0.18	[126]
Wheat	Kcb = 1.64 × NDVI − 0.12	[31]
Row vineyard	Kcb = 1.44 × NDVI−0.1	[37]
Garlic	Kcb = −1.56 × NDVI^2^ + 2.66 × NDVI − 0.08	[35]
Bell pepper	Kcb = −0.12 × NDVI^2^ + 1.45 × NDVI − 0.06	[35]
Broccoli	Kcb = −1.48 × NDVI^2^ + 2.64 × NDVI − 0.17	[35]
Lettuce	Kcb = −0.11 × NDVI^2^ + 1.39 × NDVI + 0.01	[35]
Corn	Kcb = 1.77 × SAVI + 0.02	[127]
Potato	Kcb = 1.36 × SAVI + 0.06	[32]
Sugar beet	Kcb = 1.74 × SAVI − 0.16	[34]
Row vineyard	Kcb = 1.79 × SAVI − 0.08	[37]
Cotton	Kcb = 1.74 × SAVI − 0.16	[113]
Garlic	Kcb = 1.82 × SAVI − 0.16	[113]
Olive	Kcb = 1.59 × SAVI − 0.14	[113]
Mandarin	Kcb = 0.99 × SAVI − 0.09	[113]
Peach	Kcb = 1.29 × SAVI − 0.12	[113]
Apple trees	Kcb = 1.82 ± 0.19 × SAVI − 0.07 ± 0.06	[39]

**Table 2:** Relevant aspects of the web-GIS-based decision support systems analyzed in the text. IRRISAT, Irrigation assisted by Satellite; TOP-SIMS, Terrestrial Observation and Prediction System Terrestrial Observation and Prediction System; IrriSat-SMS, Irrigation Water Management by Satellite and SMS; SPIDER, System of Participatory Information, Decision support and Expert knowledge for irrigation River basin water management; EEFlux, Earth Engine Evapotranspiration Flux.

	IRRISAT	TOP-SIMS	IrriSat-SMS	SPIDER	EEFlux
Accessibility	User and password	Open	Accessible with Gmail account	User and password	Open
Base maps	Google Satellite/Open street maps	Google Satellite/Google Terrain	Google Satellite/Google Terrain	Google Maps/Open street map	Google Maps/Open street maps
Processing time	24 h after delivery	-	Automatic after delivery	24 h after delivery	-
RS-based approach	RS-PM	Kcb-VI	Kcb-VI	Kcb-VI	METRIC
Most elaborated product	Maps of irrigated areas, LAI, CWR	Maps of Kcb and crop transpiration	Water balance components	Maps of Kcb, ETo and CWR	Actual ET, accounting for water stress
Coverage	Campania Region (Italy); Bookpournong (Australia)	California	Global, ETo available for the east of Australia	Pilot areas, 400,000 km^2^ for the largest project.	Global
Period covered	2007–2016	2010–2016	2014–2016	2013–2016	-
Dedicated App	No	No	No	Yes	No
